# Semiquantitative Proteomic Research of Protein Plasma Profile of Volunteers in 21-Day Head-Down Bed Rest

**DOI:** 10.3389/fphys.2020.00678

**Published:** 2020-07-24

**Authors:** Daria N. Kashirina, Alexander G. Brzhozovskiy, Ludmila Kh. Pastushkova, Alexey S. Kononikhin, Christoph H. Borchers, Evgeny N. Nikolaev, Irina M. Larina

**Affiliations:** ^1^Institute of Biomedical Problems–Russian Federation State Scientific Research Center, Russian Academy of Sciences, Moscow, Russia; ^2^CDISE, Skolkovo Institute of Science and Technology, Moscow, Russia; ^3^Department Oncology, Faculty of Medicine, McGill University, Montreal, QC, Canada

**Keywords:** bed rest, proteomics, label free analysis, blood plasma, resistive vibration exercise

## Introduction

During spaceflight, a complex of spaceflight factors impacts on the human organism (such as radiation, weightlessness, artificial habitat, etc.) causing various adaptive changes to occur in the human body, including changing of gene expression and protein synthesis. Due to certain ethical restrictions and complexity associated with the delivery of biological samples (costliness, lack of free space on board the descent module), there is a relatively small number of the opportunity to study these factors, one of which is a ground-based model experiment such as head-down bed rest (HDBR). HDBR is a ground-based experiment that is widely used to model the microgravity effects of spaceflight. During HDBR, human mobility is limited by strict bed rest; the longitudinal axis of the body is tilted so that the head is below the legs. Under these conditions, interdependent reactions from various body systems occur. It is noted that as a result of this, adaptation mechanisms are activated, in particular of the cardiovascular, endocrine, central nervous, and peripheral nervous systems (Larina et al., 1999; Batchu et al., 2015). The negative effect of HDBR on the cardiovascular system is realized with a decrease in the volume of circulating plasma and redistribution of vasoconstrictor and pressor influences in the vessels of various regions of the body (Baranov et al., 2016). HDBR often leads to orthostatic intolerance. Orthostatic intolerance develops as a result of hypovolemia, increased vasoconstriction response of cerebral vessels, decreased sensitivity to vasoconstrictors of resistance vessels, and decreased myocardial contractility.

For deceleration of the adaptive changes to spaceflight conditions, as well as for the astronaut's organism preparation for the ground conditions, a set of recovery measures and trainings is used, the effectiveness of which can be estimated at the molecular level using the proteomic approach in model experiments. Numerous bed rest studies of various durations have been conducted without or with specific countermeasures, such as exercises and vibrations (Pavy-Le Traon et al., 2007). The studies were mainly aimed at studying changes in the expression of muscle proteins and analyzing of the signaling pathways for proteins changed under the influence of chronic unloading.

It was shown that resistive vibration exercises (RVEs) preserved the mass and diastolic volume of the left ventricle and the contractility of the heart during HDBR (Greaves et al., 2019). It was demonstrated that this countermeasure prevented changes in the autonomic nervous system associated with deconditioning of the cardiovascular system (Coupé et al., 2011), though RVE did not effectively prevent orthostatic intolerance (Coupé et al., 2011).

Nowadays, proteomic methods allow us to expand our understanding of the mechanisms of the adaptive processes occurring under the influence of various extreme conditions. Understanding protein expression is the key to deciphering the mechanisms of action of microgravity and ultimately to finding effective countermeasures to prevent negative changes. Untargeted proteomic approach based on mass spectrometry allows to study a huge amount of proteins in a sample (the dynamic range of plasma protein concentrations is up to 10–11 orders of magnitude) (Wu and Han, 2006) and to find proteins specific for the studied effects.

Moriggi et al. (2010) revealed a substantial downregulation of proteins involved in aerobic metabolism when investigated in biopsies of the calf soleus (SOL) and the vastus lateralis (VL) in a 55-days bed rest experiment. They also showed that proteins involved in anaerobic glycolysis were upregulated when RVE countermeasures were used. Proteomic analysis of biopsy samples from the volunteers of bed rest showed that both RVE and resistive exercise led to a differential regulation of various skeletal muscle proteins (Salanova et al., 2014). RVE has been shown to prevent muscle atrophy and ultrastructural muscle changes in chronic unloading.

Dillon et al. (2019) showed that HDBR led to alterations in the expression and phosphorylation of several metabolic and structural proteins. Inclusion of exercise modulated the proteomic responses toward cellular reorganization.

Salanova et al. (2015) showed that 60 days of bed rest resulted in gene transcription and proteomic changes in the human soleus muscle. These changes were associated with various key metabolic pathways (glycolysis, tricarboxylic acid cycle, oxidative phosphorylation, lipid metabolism) and functional contractile structures. It was demonstrated that RVE countermeasures helped to reduce key signs of maladaptation and atrophy, as well as maintain normal skeletal muscle quality after chronic unloading in bed rest (Salanova et al., 2015). So, the proteomics of muscles have already been studied well, but no one has analyzed proteome changes in the blood. This can provide additional information about changes at the system level.

A blood test using a panoramic proteomic method will help to identify the effects of HDBR in a comprehensive manner, without reference to any one organ or tissue. All tissues are washed with blood, so any physiological changes are reflected in changes in the proteomic composition of the blood. Thus, the aim of the study was to compare proteomic data on the effects of HDBR with or without countermeasures on the human body. To identify protein changes, estimate the rehabilitation measures' effectiveness, and evaluate their contribution at the molecular level, a semiquantitative proteomic analysis of blood plasma samples obtained from eight volunteers who participated in HDBR with and without RVEs was performed.

## Materials and Methods

### Head-Down Bed Rest Design

Eight healthy men (20–45 years old) participated in the experiment with HDBR for 21 days with an angle of inclination of the longitudinal axis of the body relative to the horizontal position of 6°. Volunteers did not suffer from any orthopedic, musculoskeletal, and cardiovascular diseases, while there was neither excess weight nor chronic or acute diseases.

HDBR was organized by the Institute of Space Medicine and Physiology (MEDES-IMPS) in Toulouse, France, and supported by the French Spatial Agency. Samples were collected 1 day before the experiment and on the 21st day of HDBR. The subjects were in controlled conditions of life, and the diet was balanced. The volunteers were not permitted to get up or to sit up during the experiment.

All the volunteers participated in the control session of HDBR without countermeasures and in the second session with RVEs comprising of squats, single leg heel, and bilateral heel raises. Between sessions, the break was 4 months.

For exercises, a special vibration platform (Galileo® Fitness, Novotec, Germany) with an angle of inclination of 6°, combined with a training device from Novotec Medical (Pforzheim, Germany), was used. The training was carried out twice a week with an interval of 3–4 days. The first workout was on the second day of the HDBR.

Physical trainings were as follows: the warm-up consisted of bilateral squats with a knee angle from 10 to 90° for 8 s with eight repetitions; bilateral squats with a knee angle from 10 to 90° for 8 s with 10 repetitions; elevations of the heel of one leg carried out from maximal dorsiflexion to maximal plantar flexion as quickly as possible until exhaustion, and the same bilateral heel raises. The vibration frequency during exercise was 24–26 Hz with an amplitude of 8 mm. More detailed characteristics of volunteers, medical examinations, and physical training design have been described previously (Kermorgant et al., 2019).

This study (registered number: 2012-A00337-36) was carried out with the recommendations of the Ethics Committee (CPP Sud-Ouest Outre-Mer I). The protocol of the experiment was approved by the French Health Authorities. All volunteers gave written informed consent in accordance with the Declaration of Helsinki.

### Blood Sampling

Blood samples were taken from a vein in the cubital fossa and were harvested in commercial Monovette tubes (SARSTEDT, Germany) containing EDTA as an anticoagulant. Immediately after collection, the samples were centrifuged, and the obtained plasma was frozen at −80°C and stored before further sample preparation for light chromatography (LC)–mass spectrometry (MS) analysis.

### Light Chromatography–Tandem Mass Spectrometry Proteomic Analysis

To prepare for proteomic analysis, 10 μl of blood plasma was depleted using Top 12 columns (Pierce). The samples were prepared via the filter-aided sample preparation (FASP) (Wiśniewski et al., 2009) using Amicon Ultra centrifugal 10-kDa filter devices. Protein mixture was reduced using 0.1 mol/L dithiothreitol (DTT) in buffer containing 8 mol/L urea and 0.2 mol/L Tris (pH 8.5), alkylated with 0.55 mol/L iodoacetamide in buffer containing 8 mol/L urea, and 0.1 mol/L Tris (pH 8.5) and digested using trypsin with a final concentration of 1:100 enzyme:protein (w/w) in 0.05 mol/L ammonium bicarbonate (17 h, 37°C).

The resulting peptide mixture was analyzed in triplicate using LC-MS method based on a nano-HPLC Dionex UltiMate 3000 system (Thermo Fisher Scientific, USA) and a timsTOF Pro (Bruker Daltonics, Germany) equipped with a nanospray ion source (positive ion mode, 1,600 V). A C18 capillary column (75 μm × 50 cm, C18, 3 μm, 100 A) (Thermo Fisher Scientific, USA) was used to separate peptides at a flow rate of 0.3 μl/min by gradient elution from 3 to 90% of phase B during 120 min. The mobile phase A consisted of 0.1% formic acid in water and mobile phase B consisting of 0.1% formic acid in acetonitrile.

### Data Analysis

LC-MS data were searched using a MaxQuant software search engine to identify proteins from the human SwissProt database. The following parameters were used: enzyme-trypsin; missed cleavage-2; taxonomy-Human; fixed modifications-Carbamidomethyl (C); variable modifications-Oxidation (M), Acetylation (N-term); peptide tolerance ± 10 ppm; MS/MS (fragments) tolerance ± 0.5 Da. A prerequisite for identifying a protein was the presence in the spectrum of at least one unique peptide of the protein. The cutoff FDR was specified to 0.01. For semiquantitative analysis, the “no label” method with the additional “match between the runs” option was used in Perseus software package that generated normalized label-free quantification (LFQ) intensities of peptides according to the algorithms described by Cox et al. (Tyanova et al., 2016). Analysis of proteomic changes was performed using logarithmized LFQ intensities. More data on analysis parameters can be found in the article (Brzhozovskiy et al., 2019).

For identification of significantly changed proteins in two sessions of HDBR, a two-sample Welch's *t*-test (*p* < 0.05) with Benjamini–Hochberg correction was used. The String web resource (v 11.0) was used to analyze proteins with a significantly changed concentration to identify GO biological processes that were reliably presented for this set of proteins. Only associations with *p* < 0.05 were included in the table. From similar processes, more general ones were selected, which were more reliable. The mass spectrometric data were uploaded to the ProteomeXchange Consortium through the PRIDE partner repository with the dataset identifier PXD013305.

### Comparative Plasma Proteome Profiling

Using MaxQuant and Perseus programs, 239 proteins were quantified in blood plasma samples of volunteers of both HDBR sessions. By using the statistical parameters reported in the *Materials and Methods* section, we recovered 33 proteins whose peak intensities significantly differed between background and 21st day of HDBR without the use of countermeasures (Table 1). Concentrations of 20 proteins were increased, while 13 proteins were decreased. According to the Gene Ontology (GO) database, most of these proteins were involved in the regulation of proteolysis, complement activation, acute inflammatory response, defense response, response to stress, fibrinolysis, blood coagulation, etc. (Table 2).

**Table 1:** Proteins are significantly different from the summary background during head-down bed rest without countermeasures (HDBR) and with resistive vibration exercises (HDBR+RVE).

**Gene names**	**Protein names**	**HDBR**			**HDBR+RVE**		
		***p*-value**	**Welch's** ***t*****-test difference**		***p*-value**	**Welch's** ***t*****-test difference**	
IGKC	Ig kappa chain C region	1.41E-03	−3.0	↓			
AMBP	Alpha-1-microglobulin	2.56E-04	−1.4	↓	1.62E-08	2.1	↑
F2	Prothrombin	1.48E-05	−1.3	↓			
AFM	Afamin	1.15E-05	−0.9	↓			
C6	Complement component C6	8.05E-04	−0.7	↓			
GPX3	Glutathione peroxidase 3	8.41E-03	−0.6	↓			
CFI	Complement factor I	7.42E-03	−0.6	↓			
BCHE	Cholinesterase	3.87E-03	−0.5	↓			
AGT	Angiotensinogen	3.47E-03	−0.5	↓			
CP	Ceruloplasmin	2.53E-05	−0.5	↓			
SERPINC1	Antithrombin-III	4.62E-05	−0.5	↓			
PCYOX1	Prenylcysteine oxidase 1	3.98E-03	−0.4	↓			
MCAM	Cell surface glycoprotein MUC18	1.95E-03	−0.2	↓			
SERPINF2	Alpha-2-antiplasmin	2.32E-03	0.4	↑			
APOE	Apolipoprotein E	2.42E-03	0.4	↑			
CPN1	Carboxypeptidase N catalytic chain	1.98E-03	0.4	↑			
C4A	Complement C4-A	1.36E-03	0.4	↑			
C5	Complement C5	1.41E-03	0.4	↑			
ITIH4	Inter-alpha-trypsin inhibitor heavy chain H4	1.96E-03	0.5	↑			
ITIH2	Inter-alpha-trypsin inhibitor heavy chain H2	2.51E-04	0.5	↑			
PROS1	Vitamin K-dependent protein S	2.37E-03	0.5	↑			
SERPIND1	Heparin cofactor 2	3.78E-04	0.5	↑	3.19E-03	−0.4	↓
SERPINA4	Kallistatin	1.26E-03	0.6	↑			
HP	Haptoglobin	4.67E-03	0.7	↑			
C1QB	Complement C1q subcomponent subunit B	2.36E-06	0.7	↑			
IGHG1	Ig gamma-1 chain C region	4.65E-03	0.7	↑			
FGA	Fibrinogen alpha chain	2.54E-03	0.7	↑	4.38E-03	0.7	↑
FGG	Fibrinogen gamma chain	4.68E-05	0.7	↑			
FGB	Fibrinogen beta chain	2.07E-06	0.8	↑			
C1QC	Complement C1q subcomponent subunit C	5.59E-08	0.8	↑			
APOM	Apolipoprotein M	1.05E-05	0.9	↑			
AZGP1	Zinc-alpha-2-glycoprotein	5.35E-04	0.9	↑			
IGHG2	Ig gamma-2 chain C region	2.24E-03	1.6	↑			
APOA1	Apolipoprotein A-I				4.03E-05	−0.6	↓
MST1	Hepatocyte growth factor-like protein				4.96E-03	0.5	↑
ECM1	Extracellular matrix protein 1				2.73E-04	0.5	↑
C1S	Complement C1s subcomponent				1.86E-04	0.5	↑
ITIH3	Inter-alpha-trypsin inhibitor heavy chain H3				1.27E-03	0.5	↑
KLKB1	Plasma kallikrein				6.88E-03	0.5	↑
CFB	Complement factor B				3.58E-05	0.6	↑
GPLD1	Phosphatidylinositol-glycan-specific phospholipase D				1.11E-03	0.6	↑
ATRN	Attractin				2.19E-04	0.6	↑
PLG	Plasminogen				9.26E-03	0.9	↑
GC	Vitamin D-binding protein				7.73E-03	1.2	↑

**Table 2:** Comparison of the biological processes enriched in two sessions of head-down bed rest (HDBR).

**Term ID**	**Term description**	**HDBR**				**HDBR+RVE**			
		**Observed gene count**	**Background gene count**	**FDR**	**Proteins**	**Observed gene count**	**Background gene count**	**FDR**	**Proteins**
GO:0030162	Regulation of proteolysis	18	742	1.27e-15	AGT, AMBP, APOE, C1QB, C1QC, C4A, C5, C6, CFI, CPN1, F2, ITIH2, ITIH4, PROS1, SERPINA4, SERPINC1, SERPIND1, SERPINF2	8	742	9.18e-06	AMBP, C1S, CFB, ECM1, GPLD1, ITIH3, KLKB1, SERPIND1
GO:0030449	Regulation of complement activation	9	52	7.04e-14	C1QB, C1QC, C4A, C5, C6, CFI, CPN1, F2, PROS1	2	52	0.0072	C1S, CFB
GO:0002673	Regulation of acute inflammatory response	9	92	3.63e-12	C1QB, C1QC, C4A, C5, C6, CFI, CPN1, F2, PROS1	3	92	0.0013	C1S, CFB, KLKB1
GO:0031347	Regulation of defense response	14	676	3.67e-11	AGT, APOE, C1QB, C1QC, C4A, C5, C6, CFI, CPN1, F2, FGA, FGB, FGG, PROS1	5	676	0.0020	APOA1, C1S, CFB, FGA, KLKB1
GO:0080134	Regulation of response to stress	17	1,299	4.84e-11	AGT, AMBP, APOE, C1QB, C1QC, C4A, C5, C6, CFI, CPN1, F2, FGA, FGB, FGG, PROS1, SERPINC1, SERPINF2	7	1,299	0.00073	AMBP, APOA1, C1S, CFB, FGA, KLKB1, PLG
GO:0042730	Fibrinolysis	6	21	1.46e-10	F2, FGA, FGB, FGG, PROS1, SERPINF2	3	21	9.81e-05	FGA, KLKB1, PLG
GO:0051246	Regulation of protein metabolic process	21	2,668	1.79e-10	AGT, AMBP, APOE, C1QB, C1QC, C4A, C5, C6, CFI, CPN1, F2, FGA, FGB, FGG, ITIH2, ITIH4, PROS1, SERPINA4, SERPINC1, SERPIND1, SERPINF2	11	2,668	2.11e-05	AMBP, APOA1, C1S, CFB, ECM1, FGA, GPLD1, ITIH3, KLKB1, MST1, SERPIND1
GO:0050776	Regulation of immune response	13	873	6.89e-09	AMBP, C1QB, C1QC, C4A, C5, C6, CFI, CPN1, F2, FGA, FGB, FGG, PROS1	7	873	0.00013	AMBP, APOA1, C1S, CFB, ECM1, FGA, GPLD1
GO:0002576	Platelet degranulation	7	129	4.24e-08	FGA, FGB, FGG, ITIH4, PROS1, SERPINA4, SERPINF2	5	129	1.11e-05	APOA1, ECM1, FGA, ITIH3, PLG
GO:0097746	Regulation of blood vessel diameter	6	129	1.31e-06	AGT, APOE, FGA, FGB, FGG, SERPINF2				
GO:0007596	Blood coagulation	7	288	5.23e-06	F2, FGA, FGB, FGG, PROS1, SERPINC1, SERPIND1	4	288	0.0013	FGA, KLKB1, PLG, SERPIND1
GO:0042060	Wound healing	8	461	7.47e-06	F2, FGA, FGB, FGG, MCAM, PROS1, SERPINC1, SERPIND1				
GO:0009611	Response to wounding					5	547	0.0011	APOA1, FGA, KLKB1, PLG, SERPIND1
GO:0006810	transport	19	4,130	1.39e-05	AFM, AGT, AMBP, APOE, APOM, AZGP1, CFI, CP, F2, FGA, FGB, FGG, HP, ITIH4, PCYOX1, PROS1, SERPINA4, SERPINC1, SERPINF2	8	4,130	0.0257	AMBP, APOA1, ECM1, FGA, GC, GPLD1, ITIH3, PLG
GO:0045834	Positive regulation of lipid metabolic process	3	135	0.0074	AGT, APOE, F2				
GO:0046889	Positive regulation of lipid biosynthetic process					2	73	0.0115	APOA1, GPLD1
GO:0045765	Regulation of angiogenesis	3	277	0.0403	AGT, C5, C6				
GO:0010906	Regulation of glucose metabolic process					2	100	0.0188	GPLD1, MST1
GO:0051346	Negative regulation of hydrolase activity					5	438	0.00071	AMBP, APOA1, ECM1, ITIH3, SERPIND1
GO:0043534	Blood vessel endothelial cell migration					2	26	0.0029	APOA1, GPLD1

Previously, it was shown that under the impact of + GX overloads after long-term spaceflights (186–380 days), petechial hemorrhages occur in the skin integument of the back, supposedly by a marked decrease in the tone of arterial and venous vessels (Kotovskaya et al., 2005). It was also reported that 21-days HDBR can cause hemorrhages in the tissues of the lower extremities during the test for orthostatic resistance (Ganse et al., 2013). Authors reported that this volunteer did not have avascular diseases, thrombosis, or thrombophlebitis in anamnesis; therefore, the prolonged bed rest can reduce the threshold for the formation of petechiae due to a decrease in vascular tone. Also, regarding thrombophography and thromboelastometry results, hypercoagulation does not occur during HDBR (Cvirn et al., 2015). Proteins that change their level at 21 days of HDBR (F2, FGA, FGB, FGG, PROS1, SERPINC1, SERPIND1) can be involved in the negative regulation of the coagulation process.

In the study of the effect of countermeasures used in HDBR on the protein composition of the blood, it was found that the use of a set of preventive measures modified the plasma proteome, as compared with HDBR as such. So, the total number of significantly changing proteins decreased, which indicated the clinical effectiveness of this complex of preventive measures. Thirteen proteins were determined (Table 1), the concentrations of which significantly changed at the end of HDBR with exercises. Eleven proteins increased, and two proteins decreased. These proteins, according to the GO database, were involved in such processes like regulation of proteolysis, fibrinolysis, platelet degranulation, complement activation, inflammatory response, and other processes similar to HDBR processes described above. The difference between the processes of the two sessions of HDBR was the appearance of such processes like regulation of glucose metabolic process, negative regulation of hydrolase activity, blood vessel endothelial cell migration, and disappearance of such processes like regulation of blood vessel diameter and regulation of angiogenesis in the second session of HDBR with RVE (Table 2).

During HDBR experiments, muscle mass loss occurs, based on the decrease of protein synthesis (Crucian and Sams, 2009), while there was no increase in the rate of proteolysis of myofibrils or activation of the ubiquitin–proteasome pathway of protein degradation (Ogawa et al., 2006). Changing of the level of the proteins involved in extracellular matrix (ECM) organization was registered at 21 days of HDBR. Such proteins were not changed during HDBR with RVEs; at the same time, the level of the other proteins involved in ECM organization was changed (FGA, KLKB1, PLG). This indicates that RVE can reduce the influence of the hypodynamic factor on changes in ECM remodeling and loss of muscle mass. In general, a similar, although less pronounced, response of the physiological systems of the body to the effects of HDBR is observed, despite the use of preventive measures for adverse effects. The main difference in the regulation of metabolism was a predominant effect on the processes of regulation of carbohydrate metabolism in the group with the use of preventive measures (physical activity). It is worth noting that this is a pilot study, and the identified proteins will need to be validated more carefully in the future.

## Data Availability Statement

The datasets generated for this study can be found in the mass spectrometric proteomic data have been deposited to the ProteomeXchange Consortium via the PRIDE partner repository with the dataset identifier PXD013305.

## Ethics Statement

The studies involving human participants were reviewed and approved by CPP Sud-Ouest Outre-Mer I. The patients/participants provided their written informed consent to participate in this study.

## Author Contributions

LP performed the head-down bed rest. DK and AB performed the sample preparation to mass spectrometry. AK and AB conducted mass spectrometric analysis. IL, CB, EN, and DK wrote the article. All authors contributed to the article and approved the submitted version.

## Conflict of Interest

The authors declare that the research was conducted in the absence of any commercial or financial relationships that could be construed as a potential conflict of interest.

## References

[B1] Baranov M. V. Katuntsev V. P. Shpakov A. V. (2016). A method of ground simulation of physiological effects of hypogravity on humans. Bull. Exp. Biol. Med. 160, 401–405. 10.1007/s10517-016-3181-0 26742752

[B2] Batchu S. N. Smolock E. M. Dyachenko I. A. Murashev A. N. Korshunov V. A. (2015). Autonomic dysfunction determines stress-induced cardiovascular and immune complications in mice. J. Am. Heart. Assoc. 4:e001952. 10.1161/JAHA.115.001952 25999402 PMC4599426

[B3] Brzhozovskiy A. G. Kononikhin A. S. Pastushkova L. Ch Kashirina D. N. Indeykina M. I.. (2019). The effects of spaceflight factors on the human plasma proteome, including both real space missions and ground-based experiments. Int. J. Mol. Sci. 20:3194. 10.3390/ijms20133194 31261866 PMC6651200

[B4] Coupé M. Yuan M. Demiot C. Bai Y. Q. Jiang S. Z. Li Y. Z.. (2011). Low-magnitude whole body vibration with resistive exercise as a countermeasure against cardiovascular deconditioning after 60 days of head-down bed rest. Am. J. Physiol. Regul. Integr. Comp. Physiol. 301, R1748–R1754. 10.1152/ajpregu.00234.2011 21900640

[B5] Crucian B. Sams C. (2009). Immune system dysregulation during spaceflight : clinical risk for exploration-class missions. J. Leukocyte Biol. 86, 1017–1018. 10.1189/jlb.0709500 19875627

[B6] Cvirn G. Waha J. E. Ledinski G. Schlagenhauf A. Leschnik B. Koestenberger M.. (2015). Bed rest does not induce hypercoagulability. Eur. J. Clin. Investig. 45, 63–69. 10.1111/eci.12383 25413567

[B7] Dillon E. L. Soman K. V. Wiktorowicz J. E. Sur R. Jupiter D. Danesi C. P.. (2019). Proteomic investigation of human skeletal muscle before and after 70 days of head down bed rest with or without exercise and testosterone countermeasures. PLoS ONE 14:e0217690. 10.1371/journal.pone.0217690 31194764 PMC6563988

[B8] Ganse B. Limper U. Bühlmeier J. Rittweger J. (2013). Petechiae: reproducible pattern of distribution and increased appearance after bed rest. Aviation Space Environ. Med. 84, 864–866. 10.3357/ASEM.3567.2013 23926665

[B9] Greaves D. Arbeille P. Guillon L. Zuj K. Caiani E. G. (2019). Effects of exercise countermeasure on myocardial contractility measured by 4D speckle tracking during a 21-day head-down bed rest. Eur. J. Appl. Physiol. 119, 2477–2486. 10.1007/s00421-019-04228-0 31531733

[B10] Kermorgant M. Nasr N. Custaud M. A. Navasiolava N. Arbeille P. Guinet P.. (2019). Effects of resistance exercise and nutritional supplementation on dynamic cerebral autoregulation in head-down bed rest. Front. Physiol. 10:1114. 10.3389/fphys.2019.01114 31507460 PMC6718616

[B11] Kotovskaya A. R. Wil-Williams I. F. Fomina G. A. (2005). The relationship of physiological reactions of astronauts with current overload + GX on the descent from orbit to earth with hemodynamic rearrangements in the conditions of short-term weightlessness. Aerospace Environ. Med. 39, 9–15.

[B12] Larina I. M. Popova I. A. Mikhailov V. M. Buravkova L. B. (1999). The hormonal mechanisms supporting muscle work during prolonged head-down tile hypokinesia. Fiziol Cheloveka 25, 117–124. 10822633

[B13] Moriggi M. Vasso M. Fania C. Capitanio D. Bonifacio G. Salanova. (2010). Long term bed rest with and without vibration exercise countermeasures: effects on human muscle protein dysregulation. Proteomics 10, 3756–3774. 10.1002/pmic.200900817 20957755

[B14] Ogawa T. Furochi H. Mameoka M. Hirasaka K. Onishi Y. Suzue N.. (2006). Ubiquitin ligase gene expression in healthy volunteers with 20-day bedrest. Muscle Nerve 34, 463–469. 10.1002/mus.20611 16868939

[B15] Pavy-Le Traon A. Heer M. Narici M. V. Rittweger J. Vernikos J. (2007). From space to Earth: advances in human physiology from 20 years of bed rest studies (1986-2006). Eur. J. Appl. Physiol. 101, 143–194. 10.1007/s00421-007-0474-z 17661073

[B16] Salanova M. Gambara G. Moriggi M. Vasso M. Ungethuem U. Belavý D. L. (2015). Vibration mechanosignals superimposed to resistive exercise result in baseline skeletal muscle transcriptome profiles following chronic disuse in bed rest. Sci. Rep. 24:17027 10.1038/srep17027 PMC4657004 26596638

[B17] Salanova M. Gelfi C. Moriggi M. Vasso M. Viganò A. Minafra L.. (2014). Disuse deterioration of human skeletal muscle challenged by resistive exercise superimposed with vibration: evidence from structural and proteomic analysis. FASEB J. 28, 4748–4763. 10.1096/fj.14-252825 25122557

[B18] Tyanova S. Temu T. Cox J. (2016). The MaxQuant computational platform for mass spectrometry-based shotgun proteomics. Nat. Protoc. 11, 2301–2319. 10.1038/nprot.2016.136 27809316

[B19] Wiśniewski J. R. Zougman A. Nagaraj N. Mann M. (2009). Universal sample preparation method for proteome analysis. Nat. Methods 6, 359–362. 10.1038/nmeth.1322 19377485

[B20] Wu L. Han D. K. (2006). Overcoming the dynamic range problem in mass spectrometry-based shotgun proteomics. Expert. Rev. Proteom. 3, 611–619. 10.1586/14789450.3.6.611 17181475

